# Coexistent Congenital Diaphragmatic Hernia with Extrapulmonary Sequestration

**DOI:** 10.1155/2016/1460480

**Published:** 2016-04-30

**Authors:** Nao Kawamura, Samarjeet Bhandal

**Affiliations:** Alberta Children's Hospital, Calgary, AB, Canada T3B 6A8

## Abstract

Bronchopulmonary foregut malformations are a heterogeneous but interrelated group of abnormalities that may contain more than one histologic feature. It is helpful to be familiar with the presentation and imaging features of bronchopulmonary foregut malformations presenting as a congenital mass or mass-like lesion, as imaging plays a central role in the evaluation of these lesions since, when symptomatic, clinical features are usually nonspecific. With imaging, the presence of other associated lesions can be determined, facilitating appropriate management to prevent the potential complications. We report a case of coexisting extralobar pulmonary sequestration and ipsilateral diaphragmatic hernia in a term neonate.

## 1. Introduction

Bronchopulmonary foregut malformations (BPFMs), also known as congenital lung malformations, are a heterogeneous group of disorders involving conducting airways, lung parenchyma, and pulmonary vasculature. These include bronchogenic cyst, pulmonary sequestration, congenital pulmonary airway malformation, congenital lobar overinflation, bronchial atresia, and pulmonary cyst. Combinations of these malformations are called hybrid malformations [1].

Congenital diaphragmatic hernias (CDH) are serious and life threatening anatomic lesions that are frequently associated with additional anatomic malformations [2].

Lesions may be detected by prenatal ultrasound or by fetal magnetic resonance imaging. The early diagnosis of the lesion with imaging facilitates appropriate management to prevent potential complications.

## 2. Case Report

We present a case of a term female neonate born with coexisting left-sided congenital diaphragmatic hernia and bronchopulmonary foregut malformation.

The pulmonary abnormality was first detected on routine prenatal fetal anatomic sonographic evaluation at 18-week and 4-day gestation as a uniformly hyperechoic left-sided chest mass, displacing mediastinal structures to the right.

**Figure 1:**
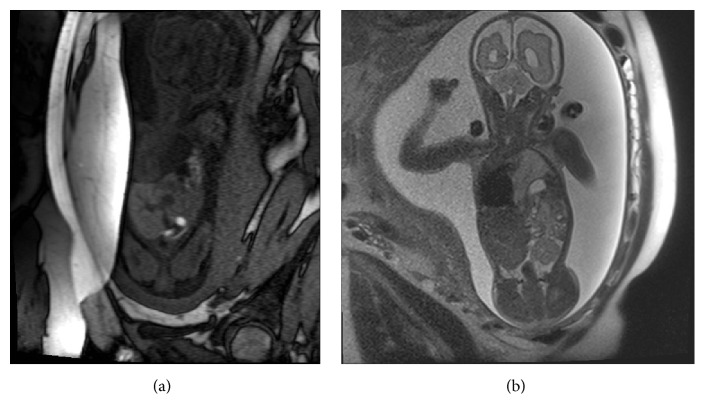
(a) T1-weighted: coronal MRI imaging at 28-week gestation showing left pulmonary bronchopulmonary foregut malformation and herniated bowel in the left hemithorax and displacement of the cardiac structures to the right side. (b) T2-weighted: coronal MRI imaging at 28-week gestation showing left pulmonary bronchopulmonary foregut malformation and herniated bowel in the left hemithorax and displacement of the cardiac structures to the right side.

Prenatal-MRI examination at 28-week and 2-day gestation (Figures 1(a) and 1(b)) confirmed a feeding vessel arising from the aorta into the centre of the pulmonary mass in keeping with systemic arterial supply. The MRI examination also diagnosed a coexisting left-sided congenital diaphragmatic hernia with stomach, multiple bowel loops, spleen, and partial liver herniation into the thoracic cavity.

**Figure 2:**
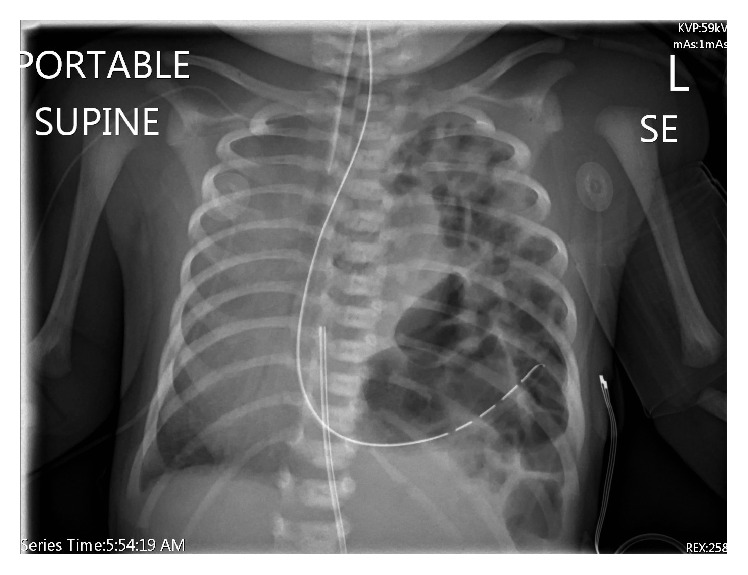
Chest radiograph showing left diaphragmatic hernia and contralateral shift of the heart and mediastinum.

The prenatal-MRI also showed markedly decreased predicted total fetal lung volumes. The infant was born at 39-week gestation by spontaneous vaginal delivery and was electively intubated at one minute of life. Initial chest X-ray (Figure 2) demonstrates herniation of bowel loops into the left chest with mediastinal shift to the right.

**Figure 3:**
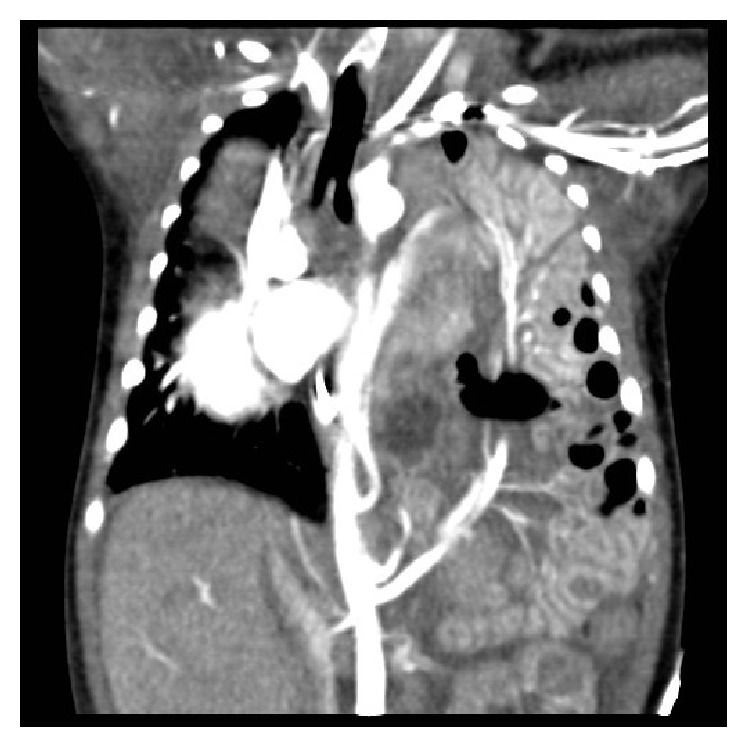
Coronal postcontrast CT image showing bronchopulmonary sequestration in a left paraspinal location. Herniated bowel loops and the superior mesenteric artery are seen in the left hemithorax. A branch from coeliac artery is seen supplying spleen, which is located in the left lower paraspinal region.

**Figure 4:**
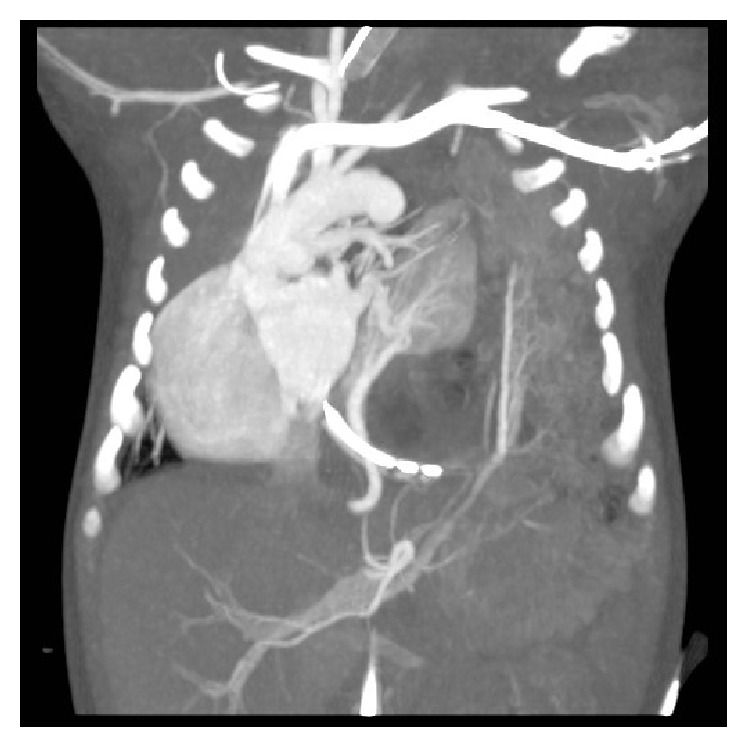
Coronal CT image showing feeding artery to sequestrated lung segment arising from the descending aorta and draining vein from the sequestration entering into left atrium.

At eight days of life, a contrast-enhanced CT examination (Figures 3 and 4) was performed which confirmed systemic arterial blood supply to the pulmonary mass. Venous drainage from the mass was to the left atrium. Left-sided congenital diaphragmatic hernia was present with herniation of small bowel loops and spleen into the thoracic cavity with marked mediastinal shift to the right.

The congenital diaphragmatic hernia was repaired at 11 days of life. Intraoperative findings were a left diaphragmatic hernia with hernia contents of the left lobe of the liver, stomach, small bowel, colon, and spleen. Once the hernia was reduced, the pulmonary sequestration was identified as being extralobar and was left in situ along the posterior mediastinum.

Her postoperative course was significant for pulmonary hypertension as a sequela to her diaphragmatic hernia. She was on oxygen supplementation and medication for approximately six months for treatment of the pulmonary hypertension which has since resolved. The patient is now over the age of two and is clinically doing well.

## 3. Discussion

BPFMs refer to a subset of pulmonary developmental anomalies that result from abnormal budding of the embryonic foregut and trachea-bronchial tree. Sequestered lung is defined as “a congenital mass of aberrant pulmonary tissue that has no normal connection with the pulmonary arteries.” The sequestrated lung is supplied by an anomalous artery arising from the aorta and its venous drainage is via the azygous system, the pulmonary veins, or the IVC [3].

Sequestrations classically have been divided into intralobar and extralobar types, in which the former accounts for 75% of all pulmonary sequestrations [4]. An intralobar sequestration is contained within the lung, has no separate pleural covering, and is intimately connected to adjacent lung. Venous drainage is usually via pulmonary veins. An extralobar sequestration has its own pleural covering and is classically described as having systemic venous drainage [5]. Our case is atypical as an extralobar sequestration with venous drainage to the left atrium.

The imaging of suspected pulmonary sequestration is directed to identification of sequestered or dysplastic lung tissue, identification of aberrant arterial and venous connections, evaluation of possible associated congenital anomalies, and assessment for diaphragmatic defects [5]. In some cases, the mass effect from a left lower lobe pulmonary sequestration is speculated to cause a protective effect in concomitant CDH, delaying the herniation of abdominal contents until after delivery and allowing prenatal lung development [6].

Other studies have reported associations of pulmonary sequestrations and CPAM with congenital diaphragmatic hernias and bronchogenic cysts [7]. The coexistence of separate anomalies in one lesion suggests that they are ends of a spectrum, rather than separate entities, and it has been suggested that it is more logical to describe all congenital cystic lung lesions as “congenital thoracic malformations” [8].

Congenital diaphragmatic hernia is characterized by incomplete formation/muscularization of the diaphragm resulting in absence or deficiency of the diaphragm. It occurs in one in 1000–2000 live births. Frequent complications are pulmonary hypoplasia and pulmonary hypertension [9]. About 50%–60% of affected individuals have isolated CDH; the remainder have complex CDH, that is, CDH occurring with additional malformations. More than 50% of cases with CDH are detected prenatally by ultrasound examination [10].

MRI is especially useful for the prenatal diagnosis of thoracic lesions that are atypical or complicated by multiple abnormalities and for assessing lung volumes. Fetal MRI can provide useful information on developmental abnormalities of the lung and thorax. The advantage of fetal MRI is that it offers operator-independent imaging in a number of different planes with excellent soft tissue contrast and a large field of view. No radiation or contrast agents are required [11].

In many cases fetal MRI affords better patient counselling, management of pregnancy and delivery, and neonatal care planning. The coexistence of a CDH may be masked by the presence of pulmonary sequestration. Therefore, infants with prenatally diagnosed lung masses, especially in the left lower lung, should be delivered at a tertiary centre and parents should be counselled about the possibility of delayed presentation of an occult CDH [6].

## Learning Objectives

1. To recognize the salient features of pulmonary sequestrations.
2. To be aware that congenital pulmonary abnormalities can be associated with congenital diaphragmatic hernia.

CanMeds competency: Medical Expert.

## Pretest

1. What are the typical distinguishing features of intrapulmonary versus extrapulmonary sequestrations?
2. Why is it important to perform tertiary level diagnostic imaging on prenatally diagnosed pulmonary chest masses?

## Posttest

1. What are the typical distinguishing features of intrapulmonary versus extrapulmonary sequestrations?
  - Both pulmonary sequestrations have aberrant aortic or systemic blood supply. An intralobar sequestration is contained within the lung, has no separate pleural covering, and is intimately connected to adjacent lung. Venous drainage is usually via pulmonary veins. An extralobar sequestration has its own pleural covering and usually has systemic venous drainage.
2. Why is it important to perform tertiary level diagnostic imaging on prenatally diagnosed pulmonary chest masses?
  - Imaging is important for characterization of the pulmonary abnormality, evaluation of possible associated congenital anomalies, and assessment for diaphragmatic defects. The antenatal and postnatal imaging features of these abnormalities guide prenatal counselling and appropriate peri- and postnatal management.
